# Effect of Low Annealing Temperature on the Critical-Current Density of 2% C-Doped MgB_2_ Wires Used in Superconducting Coils with the Wind-and-React (W&R) Method—High-Field and High-Temperature Pinning Centers

**DOI:** 10.3390/ma16186157

**Published:** 2023-09-11

**Authors:** Tomasz Czujko, Daniel Gajda, Matt Rindfleisch, Michał Babij, Andrzej Zaleski

**Affiliations:** 1 Institute of Materials Science and Engineering, Military University of Technology, Kaliskiego 2, 00-908 Warsaw, Poland; 2 Institute of Low Temperature and Structure Research, Polish Academy of Sciences (PAS), Okolna 2, 50-422 Wrocław, Poland; m.babij@intibs.pl (M.B.); a.zaleski@intibs.pl (A.Z.); 3 Hyper Tech Research, Inc., 539 Industrial Mile Rd., Columbus, OH 43228, USA; mrindfleisch@hypertechresearch.com

**Keywords:** critical-current density, MgB_2_ wires, MgB_2_ coils

## Abstract

The use of a low annealing temperature during the production of coils made from superconducting materials is very important because it reduces the production costs. In this study, the morphology, transport critical-current density (*J*_c_), irreversible magnetic field (*B*_irr_), and critical temperature (*T*_c_) of straight wires and small 2% C-doped MgB_2_ coils were investigated. The coils were made using the wind-and-react (W&R) method and annealed at various temperatures from 610 °C to 650 °C for 2–12 h. Critical-current measurements were made for both the coils and straight wires at the temperatures of 4.2 K, 20 K, 25 K, and 30 K. During our research study, we determined the process window that provides the best critical parameters of the coils (annealing at a temperature of 650 °C for 6 h). Moreover, we observed that small coils made with unreacted MgB_2_ wire and then annealed had morphology and critical parameters similar to those of straight 2% C-doped MgB_2_ wires. Moreover, small-diameter bending of 20 mm and 10 mm did not lead to transverse cracks, which can cause a large reduction in *J*_c_ in the coils. This indicates that the processes of optimization of thermal treatment parameters can be carried out on straight MgB_2_ wires for MgB_2_ superconducting coils.

## 1. Introduction

Currently, they are many different superconducting materials, e.g., high-temperature superconductors [1], including iron-based superconductors [2], NbTi [3], and Nb_3_Sn [4]. However, now, it seems that the MgB_2_ material is the most promising one for the production of coils because it consists of cheap components (low price of wires) and is characterized by high critical temperature (39 K liquid hydrogen, cryocooler), low anisotropy (wires made using the powder-in-a-tube method), and high upper critical field [5,6]. Most often, superconducting coils are made with MgB_2_ wire by using two methods: react-and-wind (R&W) and wind-and-react (W&R) methods [5]. During the production of a MgB_2_ coil using the R&W method, many difficulties are encountered, e.g., a small bending radius (e.g., 130 mm [5]) and damage to the structure of the MgB_2_ material during the winding process. This leads to a decrease in the transport critical-current density. These disadvantages result from the high hardness and brittleness of the MgB_2_ material [6]. Superconducting coils are made with long sections of MgB_2_ wire (more than 1 km). One fault in the MgB_2_ wires causes a significant reduction in the magnetic field of the coils or the complete inability to use the coils. Conducted tests showed that the wind-and-react (W&R) method is much better than the react-and-wind (R&W) method, because it allows for a large bending radius of the wires (diameter 7.5 mm [5]) and reduces the amount of damage to the MgB_2_ material, Nb barrier, and wire shields during the coil-winding process [7,8,9,10,11]. Studies also showed that annealing MgB_2_ coils made with W&R methods under high isostatic pressure does not lead to damage to the structure of the MgB_2_ material [12]. This allows for the production of high-field MgB_2_ superconducting coils.

Currently, they are two types of MgB_2_ wires: ex situ ones, where MgB_2_ powder is synthesized and used as a raw material in a powder-in-tube conductor, and in situ ones, where Mg and B are raw materials placed in powder-in-tube (PIT) wires with a diffusion barrier [6,13]. The barrier is very important, because it significantly reduces the amount of impurities. This allows one to obtain high critical-current density (*J_c_*). Heat treatment depends on the type of MgB_2_ wire. In the case of ex situ MgB_2_ wires, annealing treatment causes the sintering of powder grains [6,13], while for in situ MgB_2_ wires, the purpose of heat treatment is to produce a reaction between Mg and B to form MgB_2_ [6,13]. The results presented by Tomsic, Rindflesich, et al. [13] showed that the continuous-tube-forming and -filling (CTFF) methods are very important because they allow for the production of very long in situ MgB_2_ wires with length of over 1 km, which are necessary for superconducting-coil manufacturing.

Sumption et al. [7] annealed MgB_2_ coils made with the W&R method at 675 °C/30 min for monofilamentary strands and 700 °C/20 min for multifilamentary strands. On the other hand, Serquis et al. [9] carried out the process of heating a MgB_2_ coil at 900 °C under the pressure of 200 MPa. Sumption et al. [11] annealed superconducting MgB_2_ coils in the temperature range of 650–800 °C. In MgB_2_ wires made with the powder-in-tube (PIT) technique, Mg has a low melting point, 650 °C [14]. This allows for the thermal treatment of MgB_2_ coils made with the W&R method to be performed at much lower temperatures. A low annealing temperature has many advantages, e.g., it reduces the cost of heat treatment of the coil; reduces distortions (deformations) on the coil turns, especially for coils made of several-kilometer-long wire; increases the homogeneity of the MgB_2_ superconducting material in the wire; and reduces the influence of the temperature gradient on the process of superconducting-phase formation. A low annealing temperature is especially important for multicomponent wires (Monel sheath, Nb barrier, Cu heat matrix, and MgB_2_ core), as it reduces the amount of stress between the individual materials and the wire and prevents damage to the wires. Tanaka et al. [8] carried out annealing in the temperature range of 600–650 °C but obtained low critical-current density, approximately 15 A/mm^2^, at 10 T and high critical-current density, about 380 A/mm^2^ °C, at 6 T at 4.2 K.

Earlier results of Ref. [6] showed that the MgB_2_ material has a surface dominant pinning mechanism, e.g., voids. Surface pinning centers allow to obtain high *J*_c_ in low magnetic fields. Further studies showed that doping allowed for an increase in *J*_c_ in middle and high magnetic fields [6]. Currently, many admixtures have been used for MgB_2_ wires and bulks, e.g., SiC [15], rare earths [16], rare earths and carbon [17], copper [18], carbon nanotubes [15,19], graphene [20], and TiB_2_ [21]. However, studies have shown that the admixture of carbon most effectively increases the critical parameters [22,23]. Intensive research is being carried out to obtain high-field pinning centers and middle- and high-temperature pinning centers. These studies are very important for wind-and-react (W&R) MgB_2_ superconducting coils. Transport measurements made for NbTi wires showed that there are three types of structural defects (pinning centers) that strongly trap the vortex lattice in different ranges of high magnetic fields, namely, dislocations, lattice substitutions, and intragrain inclusions [24]. Previous studies have shown that C-for-B substitutions [22,25] and the HIP process [26] can create high-field pinning centers in MgB_2_ wires. Current research has not indicated the range of operation of high-field and high-temperature pinning centers in MgB_2_ wires. This knowledge is necessary to optimize the production process of MgB_2_ coils, especially wind-and-react (W&R) coils.

The main goal of our research was to show the effect of low-temperature heat treatment and bending on the 2% C-doped MgB_2_ material structure and Nb diffusion barrier in straight wires and coils. In addition, our research shows the effect of low annealing temperature and bending on transport critical-current density in the temperature range from 4.2 K to 30 K. Additionally, our results indicate that the peak of *J*_c_ at 20 K may appear in 2% C-doped MgB_2_ wires. These results indicate the types of pinning centers that strongly trap the vortex lattice in high magnetic fields.

## 2. Materials and Methods

MgB_2_ wires were made using the continuous-tube-forming and -filling (CTFF) technique with an admixture of 2 at% nanocarbon [13]. Pavezyum (Pavezyum Advanced Chemicals, Istanbul, Turkey) nanoboron (250 nm) of high purity was used in the wire. The 0.84 mm diameter wire was made with a Monel sheath and a Nb barrier. The wire had a superconducting material fill factor of 15%. All samples were annealed in quartz ampoules and argon atmosphere at temperatures of 610 °C, 630 °C, and 650 °C for 2 h, 6 h, and 12 h, respectively.

Microstructure evaluation was carried out using an FEI Quanta 3D (Quanta, Hillsboro, OR, USA) field emission gun scanning electron microscope (FEG-SEM) equipped with electron backscattered and secondary electron detectors, and an energy-dispersive spectroscopy (EDS) chemical composition analyzer. Microscopic observations were conducted across entire sections of samples. All samples, before structural examinations, were subjected to the following metallographic preparation: grinding on SiC paper with granulation in sequence of 120, 240, 500, 1200, and 2400; polishing with diamond suspensions (3 µm, 1 µm, and 0.25 µm); and finally, polishing with silica suspensions of 0.1 µm and 0.06 µm.

All transport measurements were performed using the four-contact method. Critical-temperature (*T*_c_), irreversible-magnetic-field (*B*_irr_), and upper-magnetic-field (*B*_c2_) measurements were made using a physical property measurement system (PPMS) with a 9 T superconducting magnet (100 mA and 19 Hz). *B*_irr_ and *T*_c2_ were determined on the basis of the criteria of 10% and 50% (resistance in the normal state). Critical-current (*I*_c_) measurements were made using the field sweep method (constant current and rapidly increasing magnetic field) and a measuring system with a Bitter magnet with a magnetic field of 14 T at 4.2 K and using the PPMS at 20 K, 25 K, and 30 K at 9 T [27]. The critical current was determined with the criterion of 1 µV/cm. The critical-current density (*J*_c_) was determined for the surface of the superconducting MgB_2_ material. The engineering critical-current density (*J*_ec_) was determined for the entire surface of the wire (Nb barrier, Monel shield, and MgB_2_ material).

## 3. Results and Discussion

### 3.1. Structural Analysis of Straight 2% C-Doped MgB_2_ Wires and Small 2% C-Doped MgB_2_ Coils

The tightness of the Nb barrier in the 2% C-doped MgB_2_ wires was checked using the field sweep [27] and temperature sweep [28] methods and with a scanning electron microscope. Both methods showed that there were no cracks in the Nb barrier. Studies have shown that cracks in the Nb barrier significantly reduce *J_c_* in MgB_2_ wires [27]. For MgB_2_ coils made with the wind-and-react method, the tightness of the Nb barrier is very important because it allows one to obtain high *J_c_* and homogeneous structure of the MgB_2_ material in wires over a length of several kilometers.

The EDS analysis of straight wires and small coils showed that the 2% C-doped MgB_2_ material had a homogeneous distribution of Mg and B, and it had a very consistent, near-stoichiometric atomic (e.g., B—62.3–65.4%; Mg—34.6–37.7%) and weight (e.g., B—78.8–8.9%; Mg—19.1–21.2%) composition. This is very important because it allows us to obtain uniform critical parameters on long MgB_2_ wires (above 1 km). This increases the application possibilities of MgB_2_ wires.

**Figure 1:**
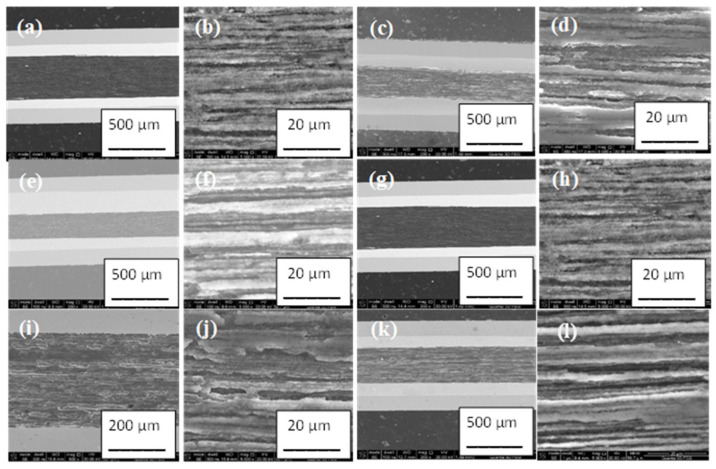
Small magnification: longitudinal sections of straight 2% C-doped MgB_2_ wires annealed (**a**,**b**) at 610 °C for 2 h; (**c**,**d**) at 630 °C for 2 h; (**e**,**f**) at 650 °C for 2 h; (**g**,**h**) at 610 °C for 12 h; (**i**,**j**) at 630 °C for 12 h; and (**k**,**l**) at 650 °C for 12 h.

Transverse cracks strongly reduce the critical-current density in MgB_2_ wires. It is clearly seen (Figure 1) that no transverse cracks appeared in the structure of the 2% C-doped MgB_2_ material in the longitudinal section of wires. The SEM results in Figure 1 showed that the 2% C-doped MgB_2_ material had a layered structure. Studies show that annealing at 610 °C, 630 °C, and 650 °C for 2 h and 6 h allowed similar morphologies of the 2% C-doped MgB_2_ material to be obtained in all samples. Further results show that a longer annealing time (12 h) at 630 °C and 650 °C improved the layered structure (Figure 1i–l). On the other hand, the long annealing time (12 h) at 610 °C did not improve the layered structure, which was similar to the structure of samples annealed for 2 h and 6 h at 610 °C, 630 °C, and 650 °C. This indicates that the synthesis reaction in the 2% C-doped MgB_2_ material at 610 °C is very slow. Our results also indicate that the reaction in the liquid state of Mg at 650 °C is not significantly faster than the synthesis reaction in the solid state of Mg at 630 °C, even over a very long time (12 h).

**Figure 2:**
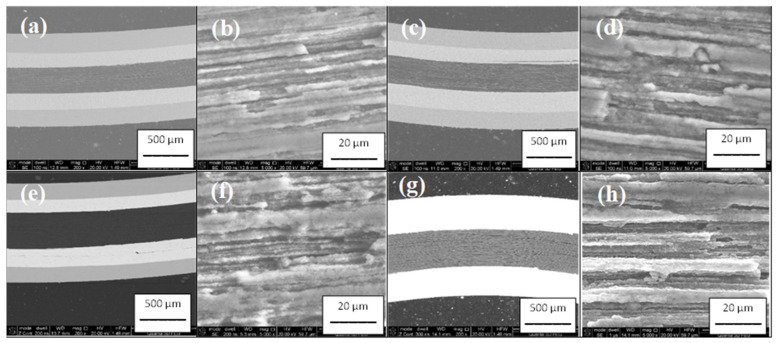
Small magnification: longitudinal sections of small 2% C-doped MgB_2_ coils annealed (**a**,**b**) at 630 °C for 2 h with d = 20 mm; (**c**,**d**) at 630 °C for 2 h with d = 10 mm; (**e**,**f**) at 650 °C for 6 h with d = 20 mm; and (**g**,**h**) at 650 °C for 6 h with d = 10 mm.

The results in Figure 2 show the longitudinal sections of 2% C-doped MgB_2_ wires on small-diameter coils of 10 mm and 20 mm. These SEM images show that the small-diameter bending of the MgB_2_ wire did not create any cracks in the structure of the 2% C-doped MgB_2_ material (Figure 2). This is very important for applications in superconducting coils. Further results show that bending did not affect the layered structure. The layered structure is very important because it creates a large number of longitudinal connections that allow a high *J*_c_ value to be obtained. In addition, this research shows that bending did not affect the synthesis reactions in solid-state and liquid-state Mg, because the structure of the 2% C-doped MgB_2_ material on the coils was similar to the structure of the 2% C-doped MgB_2_ material on straight wires (Figure 2).

**Figure 3:**
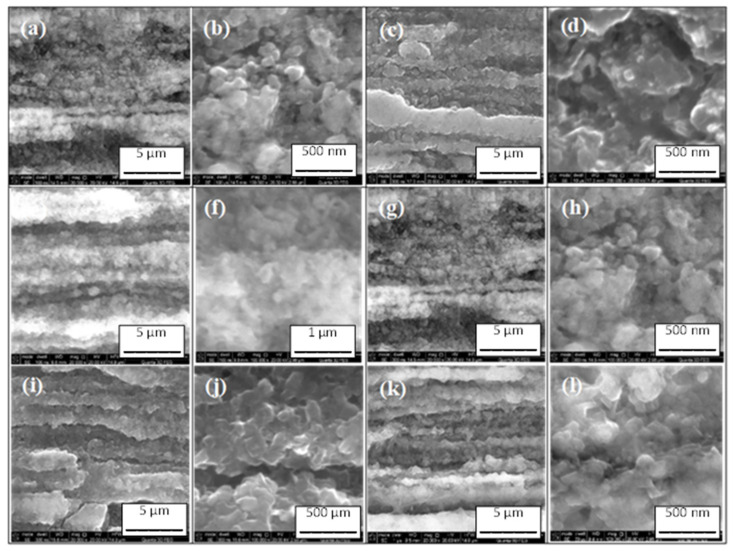
High magnification: longitudinal sections of straight 2% C-doped MgB_2_ wires annealed (**a**,**b**) at 610 °C for 2 h; (**c**,**d**) at 630 °C for 2 h; (**e**,**f**) at 650 °C for 2 h; (**g**,**h**) at 610 °C for 12 h; (**i**,**j**) at 630 °C for 12 h; and (**k**,**l**) at 650 °C for 12 h.

In Figure 3 (high magnification), we see that after annealing at temperatures of 610 °C, 630 °C, and 650 °C for 2 h and 6 h, the samples did not have cracks and had similar grain sizes. Additionally, the results in Figure 3 show that the sample annealed at 630 °C for 2 h had slightly better grain connections. The next results show that a long annealing time (12 h) and annealing temperatures of 630 °C and 650 °C significantly improved the connections among the grains (Figure 3). On the other hand, annealing at 610 °C for 12 h did not improve the connections among grains (Figure 3).

**Figure 4:**
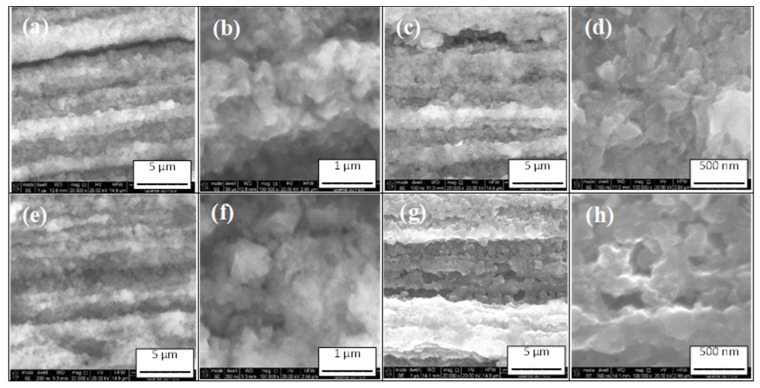
High magnification: longitudinal sections of small 2% C-doped MgB_2_ coils annealed (**a**,**b**) at 630 °C for 2 h with d = 20 mm; (**c**,**d**) at 630 °C for 2 h with d = 10 mm; (**e**,**f**) at 650 °C for 6 h with d = 20 mm; and (**g**,**h**) at 650 °C for 6 h with d = 10 mm.

Figure 4 shows that unreacted 2% C-doped MgB_2_ wire bending with small diameter and thermal treatment did not create micro- and nanocracks among grains and did not affect the size and growth rate of grains. Our research shows that the morphology of the 2% C-doped MgB_2_ material on straight and bent wires was similar (Figure 3 and Figure 4).

### 3.2. Irreversible-Magnetic-Field Analysis of Straight 2% C-Doped MgB_2_ Wires and Small 2% C-Doped MgB_2_ Coils

The results of the transport measurements showed that increasing the annealing time from 2 h to 12 h at 610 °C slightly increased *T*_c_ (from 32.2 to 32.5 K). Further results show that increasing the annealing time from 2 h to 12 h at 630 °C led to an increase in *T*_c_ from 32.5 to 33.5 K. The next results show that increasing the annealing time from 2 h to 6 h at 650 °C increased *T*_c_ from 33.5 to 35 K. A further increase in the annealing time (12 h) did not increase *T*_c_. The measurements indicate that increasing the annealing temperature from 610 °C to 650 °C for 2 h increased *T*_c_ by 1.3 K. On the other hand, increasing the annealing temperature from 610 °C to 650 °C for 12 h led to an increase in *T*_c_ by 2.5 K. Earlier studies showed that the critical temperature (*T*_c_) is dependent on stress, strain, stoichiometry, doping, pressure, coherence length, free path, and the lattice parameter [22,29]. These results indicate that a higher annealing temperature and a longer annealing time allowed us to obtain 2% C-doped MgB_2_ material with higher *T*_c_, because we obtained a material more similar to the MgB_2_ superconducting phase with a critical temperature of 39 K. The increase in *T*_c_ indicates that the process of substituting carbon for boron is not significant while heating at low temperature.

**Figure 5:**
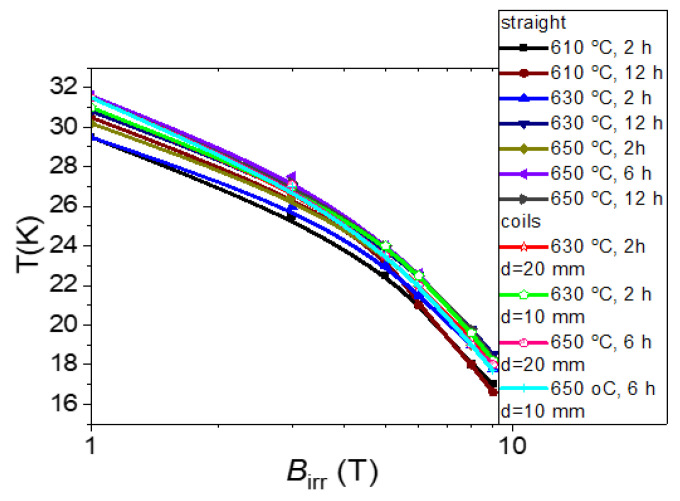
Dependence of the irreversible magnetic field on temperature for straight 2% C-doped MgB_2_ wires and small 2% C-doped MgB_2_ coils.

Previous research indicates that the irreversible magnetic field in superconducting materials depends on the pinning centers [24,30]. The results in Figure 5 show that increasing the annealing time from 2 h to 12 h at 610 °C led to an increase in *B*_irr_ in the high-temperature range (26 K to 30 K) and did not increase *B*_irr_ below 26 K.

**Figure 6:**
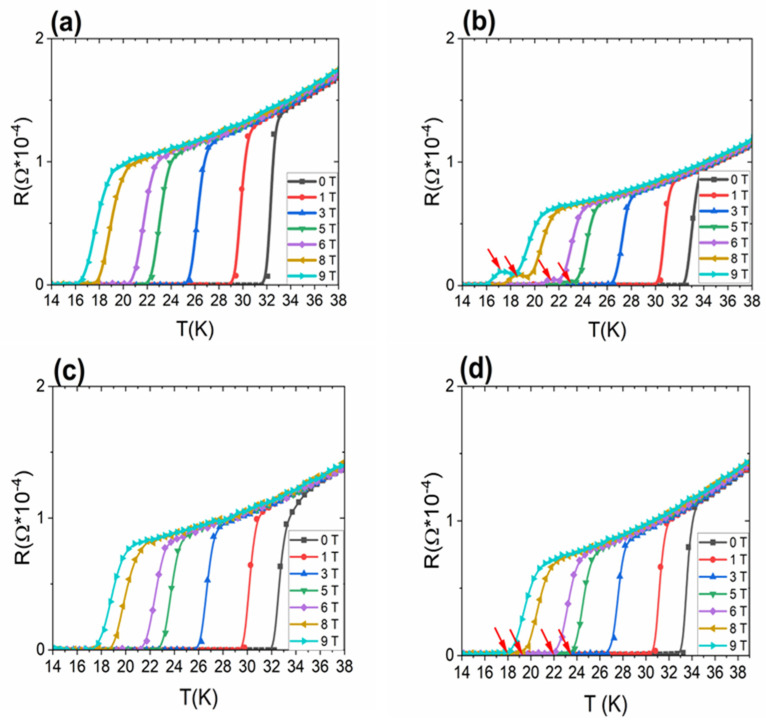
Dependence of resistance on temperature (**a**) for sample A annealed at 610 °C for 2 h; (**b**) sample G annealed at 610 °C for 12 h; (**c**) sample B annealed at 630 ^°C^ for 2 h; and (**d**) sample H annealed at 630 °C for 12 h.

The results in Figure 6a,b indicate the cause of the change in *B*_irr_ of sample G. The transport results in Figure 6b show the peak effect of resistance (red arrow) in the temperature range of 16 K to 24 K. This is the first time this effect has been observed in 2% C-doped MgB_2_ wires depending on the temperature. Previously, a similar effect was observed in NbTi wires depending on the magnetic field [24] and in MgB_2_ crystals depending on the magnetic field [31]. In NbTi wires, this effect was related to the strong trapping of the vortex lattice by different pinning centers in different ranges of magnetic fields [24]. In MgB_2_ crystals, this effect was the result of the pinning forces for the motion of vortex spots transverse to the applied field becoming larger than those for parallel motion, and the anisotropic vortex current distribution induced a reduced energy barrier for vortex nucleation at the edge perpendicular to the field [31]. The above results indicate that the resistance peak obtained is related to the low density of pinning centers close to *B*_irr_. The results in Figure 6b show, for the first time, the effect of individual pinning centers on *B*_irr_ depending on the temperature. Based on these results, we can indicate that high-field and high-temperature pinning centers (pinning centers trap vortex lattices from 25 K to 30 K) poorly trap the vortex lattice at middle temperatures and in high magnetic fields. In addition, the results indicate that high-field and middle-temperature (from 16 K to 25 K) pinning centers poorly trap the vortex lattice in high magnetic fields and at high temperatures. This is a very important measurement result, because it allows us to better understand the effect of the trapping process of the vortex lattice on structural defects. These results also show that at low annealing temperatures, high-field and low-temperature pinning centers form much more slowly than low-field and high-temperature pinning centers. Further results in Figure 6c,d show that higher annealing temperature and longer annealing time increase the number of high-field and middle-temperature pinning centers and remove the resistance peak. This suggests that high-field pinning centers may form a carbon-for-boron substitution. This process at low annealing temperatures is slow and produces a small number of pinning centers. That is why we see a resistance peak.

Further results show that increasing the annealing temperature from 610 °C to 630 °C for 2 h led to an increase in *B*_irr_ at moderate temperatures and high magnetic fields (Figure 5). This indicates that it creates more high-field and low-temperature pinning centers. An increase in the annealing time from 2 h to 12 h at an annealing temperature of 630 °C led to an increase in *B*_irr_ at low, middle, and high temperatures. This indicates that this annealing process can create low-, mid-, and high-field pinning centers. A further increase in the annealing temperature from 630 °C to 650 °C for 2 h led to an increase in *B*_irr_ at high temperatures. This indicates that it mainly forms high-temperature pinning centers. The increase in the annealing time from 2 h to 12 h at 650 °C led to a slight increase in *B*_irr_.

The next results show that small coils, after annealing at 630 °C for 2 h, had a *B*_irr_ value that was 10% higher than that of a straight 2% C-doped MgB_2_ wire from the same annealing process. Previous results showed that small coils and simple coils had the same *B*_irr_ [12]. However, these processes were performed in liquid Mg. Our heating process for J and K coils involved solid-state Mg. This may indicate that cold bending and heat treatment in the solid state of Mg can yield more pinning centers. Further results show that small coils and straight wires annealed at 650 °C for 6 h had similar *B*_irr_. This indicates that cold bending and thermal treatment in the liquid state of Mg do not affect the formation of pinning centers.

The transport measurements in Figure 6 show that our samples did not exhibit magnetoresistance. This indicates that no unreacted Mg remained in our samples after the heat treatment process. Our MgB_2_ wire was made with a Nb barrier, a Monel sheath, and a 2% C-doped MgB_2_ core. All these components have higher resistance than pure Mg. The next results show that increasing the annealing temperature from 630 °C to 650 °C led to a decrease in resistance in the normal state by approximately 15%, especially for the 2 h annealing time. This indicates that a higher annealing temperature allows for a greater number of connections among grains. Further results show that a longer heating time (2 h to 12 h) at 610 °C reduced resistance in the normal state by 22%. On the other hand, increasing the heating time (2 h to 12 h) at 630 °C reduced resistance in the normal state by approximately 15%. The next transport measurements showed that an increase in the annealing time (2 h to 6 h) for the annealing temperature of 650 °C led to a decrease in resistivity in the normal state by 30%. A longer heating time at 650 °C did not reduce resistance in the normal state. This indicates that a longer annealing time allows a greater number of connections to be obtained among grains. Moreover, the results in Figure 6 indicate that the samples had a narrow superconducting-state-to-resistive-state transition. We observed such effects for all heating times and temperatures and for small coils. This shows that the MgB_2_ wires have high homogeneity of 2% C-doped MgB_2_ material. These factors are important for the design of superconducting coils.

### 3.3. Critical-Current Density in Straight 2% C-Doped MgB_2_ Wires and Small 2% C-Doped MgB_2_ Coils

#### 3.3.1. *J_c_* and *J_ec_* at 4.2 K

**Figure 7:**
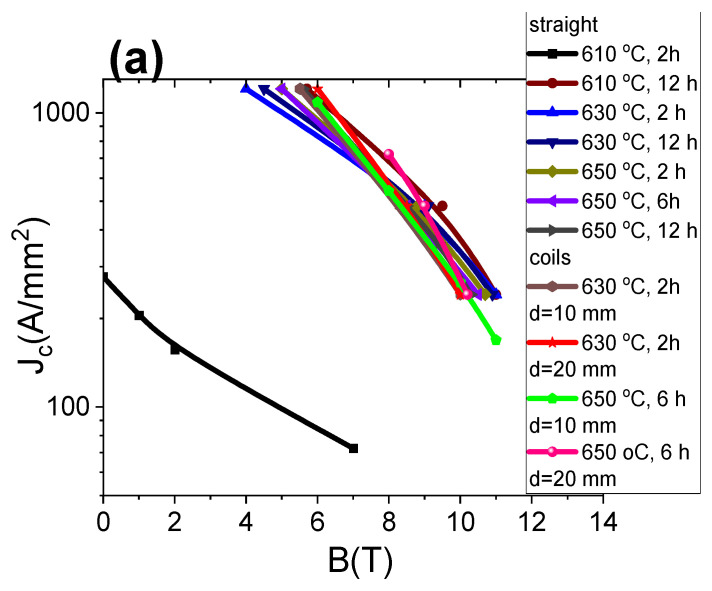

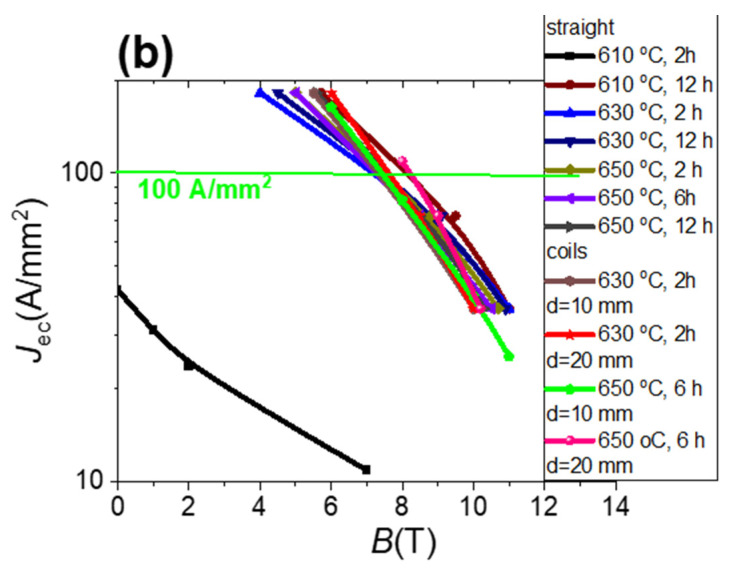
(**a**) Dependence of the critical-current density on the magnetic field; (**b**) dependence of the engineering critical-current density on the magnetic field at 4.2 K for straight 2% C-doped MgB_2_ wires and small 2% C-doped MgB_2_ coils.

The results in Figure 7a show that increasing the annealing time from 2 h to 12 h at 610 °C significantly increased the critical-current density. Further results show that an increase in the annealing temperature and annealing time did not lead to an increase in *J*_c_ at 4.2 K. Additionally, the results show that *J*_c_ at 4.2 K for small coils was the same as *J*_c_ for straight MgB_2_ wires. This indicates that the wind-and-react (W&R) method is very good, because it does not lead to the degradation of *J*_c_ even on large bends (diameter of 10 mm). Our results also indicate that low annealing temperatures can be used for annealing the coils. This is important because it reduces the costs of coil production. In addition, we obtained a very high *J*_c_ value of 400 A/mm^2^ (4.2 K in 10 T) in powder-in-tube (PIT) 2% C-doped MgB_2_ wires. The *J*_c_ value in our PIT 2% C-doped MgB_2_ wires is very similar to the *J*_c_ in wires and small coils made using internal Mg diffusion (450 A/mm^2^, 4.2 K, 10 T, 650 °C for 5 h) [32]. Our results indicate that a low annealing temperature has great potential for increasing *J*_c_ in wires and coils.

The results in Figure 7b show the engineering critical-current density (*J*_ec_) values for straight wires and small coils. *J*_ec_ was determined for the whole wire (superconducting material, Nb barrier, Monel sheath). *J*_ec_ is a very important factor in the design of superconducting coils. Currently, the application criterion for superconducting coils is a *J*_ec_ value of 100 A/mm^2^. Our results in Figure 7b show that a low annealing temperature allows one to obtain straight wires and small coils with a *J*_ec_ value of 100 A/mm^2^ at 8.2 T. This indicates that our wire can be used to produce coils with a magnetic field of 8 T.

#### 3.3.2. *J_c_* at 20 K, 25 K, and 30 K

The critical-current (*I*_c_) measurements at 20 K, 25 K, and 30 K were performed using a PPMS. The critical current was determined based on the criterion of 0.5 µV/cm, because the voltage contacts were 5 mm apart.

**Figure 8:**
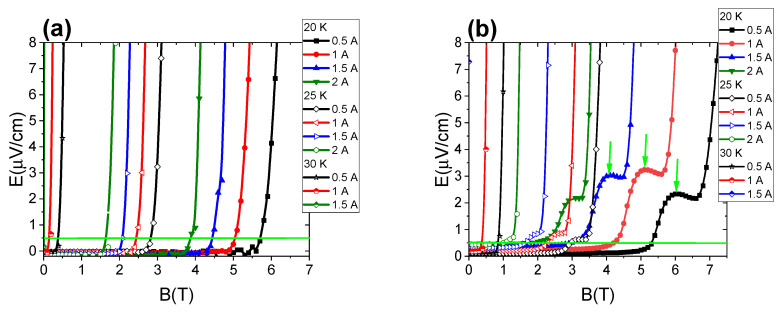

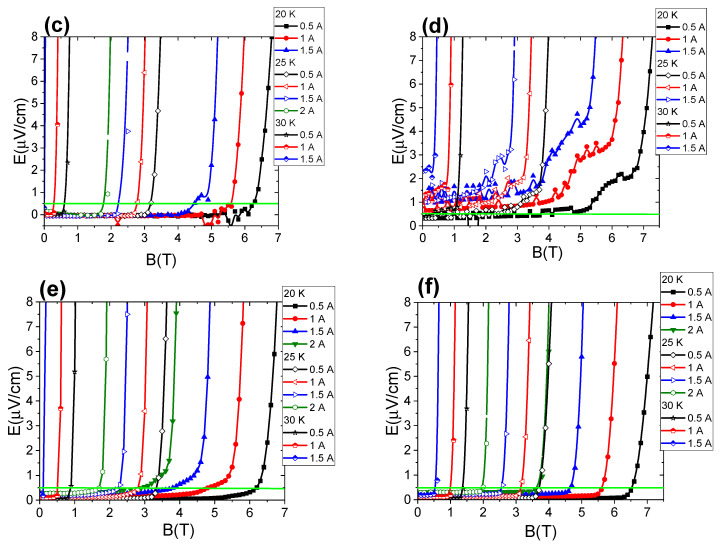
Dependence of the electric field on the magnetic field (**a**) for samples annealed at 610 °C for 2 h, (**b**) for samples annealed at 610 °C for 12 h, (**c**) for samples annealed at 630 °C for 2 h, (**d**) for samples annealed at 630 °C for 12 h, (**e**) for samples annealed at 650 °C for 2 h, and (**f**) for samples annealed at 650 °C for 6 h for various critical-current density values.

By analyzing the measurements in Figure 8a,b, we see that a longer annealing time (from 2 h to 12 h) at 610 °C led to the formation of the electric-field peak (green arrows). This peak is the greatest at 20 K and disappears with a temperature increase from 20 to 30 K.

A similar resistance peak was observed in MgB_2_ crystals by Rydh et al. [31], but this peak increased with the increase in temperature (from 23 K to 27 K).

This electric-field peak is the result of the low density of the high-field pinning centers. A similar effect was observed in NbTi wires. This effect is called peak effect. This means that we obtain a higher *J*_c_ value close to *B*_irr_ or *B*_c2_ [24] than middle and low magnetic fields. The decrease in the electric-field value may be because high-field pinning centers poorly trap the vortex lattice at middle temperatures (20 K and 25 K). Therefore, there is a smaller peak of the electric field at 25 K than at 20 K, and it decays at 30 K. This shows the properties of high-field pinning centers. In addition, the results show that a longer annealing time at 610 °C decreased *I*_c_, especially at 20 K and 25 K, and increased *I*_c_ at 30 K. This indicates that a longer annealing time is necessary to produce high-field and high-temperature pinning centers. Moreover, the results indicate that long annealing times created more pinning centers at 30 K. Figure 8a,b show that high-field pinning centers effectively increased *I*_c_ at 20 K and 25 K.

The results of transport measurements in Figure 8c,d show that increasing the annealing time from 2 h to 12 h at 630 °C led to a decrease in *I*_c_ in high magnetic fields at 20 K and 25 K and to an increase in *I*_c_ at 30 K. This indicates that a long annealing time removes high-field pinning centers, e.g., dislocations, stresses, and strains. These results also indicate that high-field pinning centers can increase *I*_c_ at 20 K and 25 K and do not increase *I*_c_ at 30 K. In addition, this also indicates that high-temperature pinning centers do not increase *I*_c_ at 20 K and 25 K. The electric field in Figure 8d in the zero magnetic field is the result of current penetration into the superconducting material. The overlapping of the curves for the same current (e.g., 0.5 A) at different temperatures and for the increase in the magnetic field is also the effect of current penetration into the superconducting material. This effect disappears for long MgB_2_ wires.

Further results show that an increase in the annealing temperature from 630 °C to 650 °C for 2 h led to a strong decrease in *I*_c_ at 20 K, a weak decrease in *I*_c_ at 25 K, and an increase in *I*_c_ at 30 K. In addition, in Figure 8e, an electric field appears before exceeding the criterion of 0.5 µV/cm, especially at 20 K and 25 K. This effect disappears at 30 K. The appearance of the electric field before exceeding the 0.5 µV/cm criterion is very unfavorable, because it significantly reduces the critical current that can be used by superconducting coils. Superconducting coils are made of several kilometers of MgB_2_ wire. Even a small electric field over a distance of 5 mm (distance of voltage contacts) leads to a large electric field on wires several kilometers long. This leads to the heating of the coil and its transition to the resistive state. In addition, based on the results in Figure 8c,e, it was seen that during the annealing process at 650 °C for 2 h, high-field pinning centers at 20 K and 25 K formed more slowly than during the annealing process at 630 °C for 2 h. This indicates that higher density of high-field pinning centers is formed during the reaction in the solid state of Mg than in the liquid state of Mg. These results also indicate that we obtained higher density of high-temperature pinning centers (30 K) during the reaction in the liquid state of Mg than in the solid state of Mg. Further results show that increasing the annealing time from 2 h to 6 h at 650 °C (Figure 8e,f) led to an increase in *I*_c_ at 20 K, 25 K, and 30 K and eliminated the effect of increasing the electric field before exceeding the criterion of 0.5 µV/cm. These results indicate that a longer annealing time at 650 °C and Mg in the liquid state allow for an increase in the density of high-field pinning centers and high-temperature pinning centers. It is very important that the electric field decays before exceeding the criterion of 0.5 µV/cm, because it allows for the use of higher *I*_c_ values in superconducting coils.

**Figure 9:**
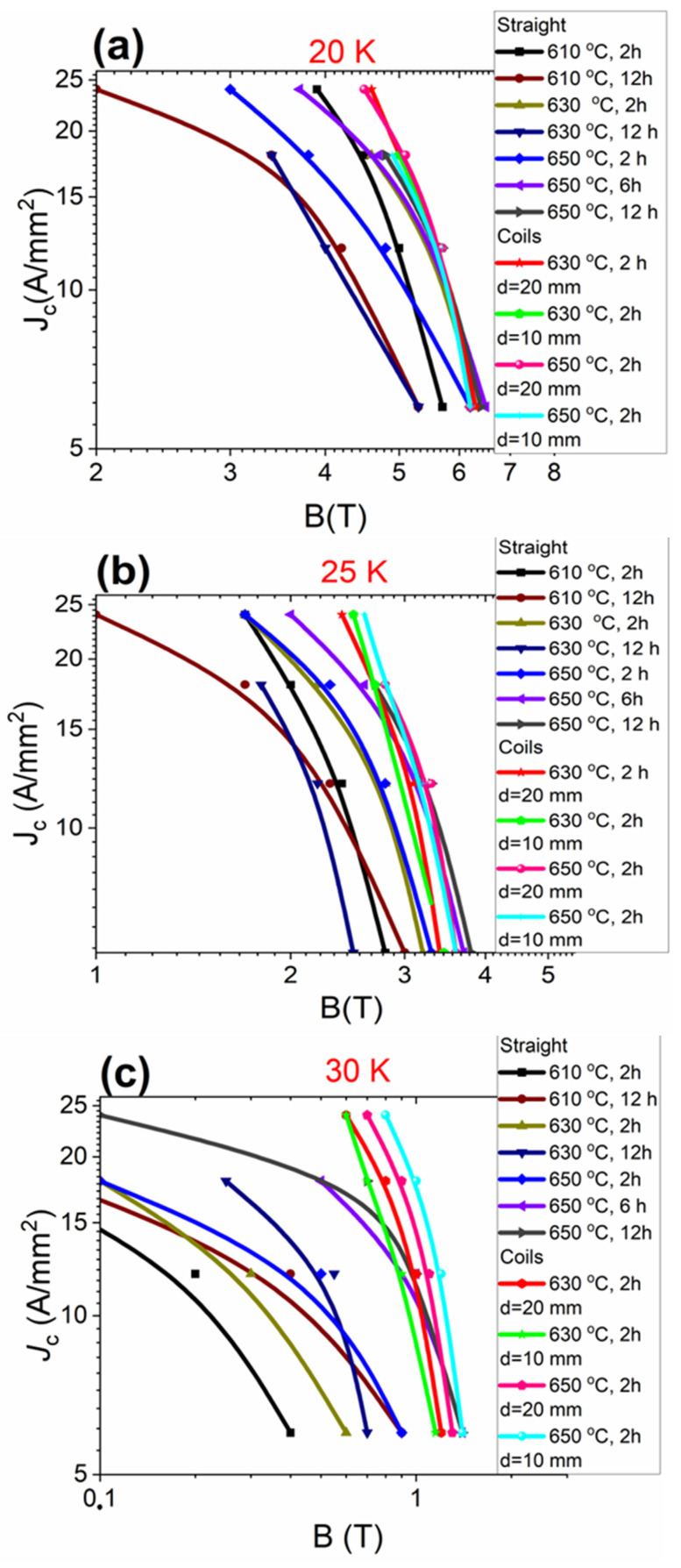
Dependence of the critical-current density on the magnetic field for straight 2% C-doped MgB_2_ wires and 2% C-doped small MgB_2_ coils at temperatures of (**a**) 20 K, (**b**) 25 K, and (**c**) 30 K.

Based on the results in Figure 9a,b, increasing the annealing time from 2 h to 12 h at 610 °C and 630 °C led to a significant decrease in *J*_c_ at 20 K and 25 K. On the other hand, increasing the annealing time from 2 h to 12 h at 650 °C led to a significant increase in *J*_c_ at 20 K and 25 K. Increasing the annealing temperature from 610 °C to 630 °C for 2 h led to an increase in *J*_c_ in high magnetic fields at 20 K and 25 K. A further increase in the annealing temperature to 650 °C caused a significant decrease in *J*_c_ at 20 K and did not influence *J*_c_ at 25 K. These results indicate that the long annealing time (12 h) at 610 °C and 630 °C and the short annealing time (2 h) at 650 °C did not allow us to obtain high density of high-field pinning centers at 20 K and 25 K. Our results also indicate that high-field pinning centers at 20 K formed much more slowly at an annealing temperature of 650 °C than at 630 °C (2 h). The results show that the *J*_c_ values at 20 K and 25 K of small coils and straight wires were similar. This is very important because it shows that the wind-and-react (W&R) method does not reduce *J*_c_ in the coil.

The results in Figure 9c show that increasing the annealing time from 2 h to 12 h at 610 °C, 630 °C, and 650 °C led to an increase in *J*_c_ at 30 K. Further results show that increasing the annealing temperature from 610 °C to 650 °C for 2 h and 12 h also led to an increase in *J*_c_ at 30 K. These results show that we can obtain a higher density of high-temperature pinning centers as a result of the longer heating time and increase in the annealing temperature. The results show that small coils after annealing at 630 °C for 2 h had a much higher *J*_c_ value at 30 K than straight wires after annealing at the same temperature. Further results indicate that coils, after annealing at 650 °C for 6 h, also had higher *J*_c_ at 30 K than straight wires after annealing at the same temperature. This result indicates that more high-temperature pinning centers formed on the coils. This effect was not visible at 20 K and 30 K. This is a very interesting and important result for future applications. This effect may be related to the change in the density of the unreacted MgB_2_ material in the wire after bending. Our research has shown that higher density of unreacted MgB_2_ material improves critical parameters at low annealing temperatures [33].

## 4. Conclusions

Our research showed that low–temperature annealing in the temperature range of 610 °C to 650 °C allowed us to obtain a structure with a similar morphology, e.g., thickness of layers, grain size and voids, and connections among grains. Studies have shown that a longer annealing time (12 h) at temperatures of 630 °C and 650 °C improves the connections between layers. Further studies showed that the structures of the 2% C-doped MgB_2_ material on straight wires and bent wires, even with small diameters, are similar. In addition, the tests showed that bending and heat treatment at low annealing temperatures did not cause transverse cracks in the 2% C-doped MgB_2_ material or the Nb barrier and Monel shield. This indicates that the wind-and-react method can be used to manufacture 2% C-doped MgB_2_ superconducting coils. Measurements showed that a longer annealing time and a higher annealing temperature allowed for an increase in the critical temperature (*T*_c_). The highest *T*_c_ measured at 35 K was obtained with the annealing process at 650 °C for 6 h. The performed studies showed that a longer annealing time was necessary to obtain high *B*_irr_ at an annealing temperature of 610 °C. On the other hand, the measurements showed that a longer annealing time at 630 °C led to a decrease in *B*_irr_. The next measurement results indicate that a longer annealing time was necessary to increase *B*_irr_ at 650 °C. The results show that the *B*_irr_ of straight wires was close to the *B*_irr_ of coils. This is very important because it indicates that bending does not cause *B*_irr_ degradation during annealing when magnesium is in a solid or liquid state.

Further results show that increasing the annealing time at 610 °C led to a significant increase in *J*_c_ at 4.2 K. Moreover, higher annealing temperatures and longer annealing times did not lead to a significant increase in *J*_c_ at 4.2 K. Our results show that low-temperature annealing can produce very high *J*_c_ at 4.2 K–400 A/mm^2^ at 10 T. Calculations show that our wires had very high engineering critical-current density, 100 A/mm^2^ at 8 T. This allowed us to produce coils with a magnetic field of 8 T.

Our results show that a low annealing temperature of 610 °C and 630 °C for 2 h allowed us to obtain high *J*_c_ at 20 K. Further studies showed that the annealing temperatures of 610 °C and 630 °C led to a significant reduction in *J*_c_ at 30 K. Further studies showed that the annealing temperature of 650 °C allowed us to obtain the highest *J*_c_ at 30 K. Transport measurements also showed that coils annealed at 630 °C had a *J*_c_ value similar to that of straight wires at 20 K and 25 K and much higher *J*_c_ at 30 K. Further results show that coils annealed at 650 °C for 6 h had a *J*_c_ value similar to that of straight wires at 20 K, 25 K, and 30 K.

Our results show that middle-temperature and high-field pinning centers increased *J*_c_ in the temperature range of 16 K to 25 K and did not influence *J*_c_ in the temperature range of 25 K to 30 K. Further measurement results show that high-temperature and high-field pinning centers increased *J*_c_ in the temperature range from 25 K to 30 K and had a weak effect on *J*_c_ in the temperature range from 16 K to 25 K. These measurement data are very important because they could allow us to better optimize the production process of superconducting coils depending on the temperature (e.g., 20 K, 30 K) at which the coil generates a magnetic field.

## Data Availability

Not applicable.
